# Risk of incident or recurrent malignancies among patients with rheumatoid arthritis exposed to biologic therapy in the German biologics register RABBIT

**DOI:** 10.1186/ar2904

**Published:** 2010-01-08

**Authors:** Anja Strangfeld, Franka Hierse, Rolf Rau, Gerd-Ruediger Burmester, Brigitte Krummel-Lorenz, Winfried Demary, Joachim Listing, Angela Zink

**Affiliations:** 1 German Rheumatism Research Centre Berlin, a Leibniz institute, Charitéplatz 1, 10117 Berlin, Germany; 2 Irisweg 5, 40489 Duesseldorf, Germany; 3 Department of Rheumatology and Clinical Immunology, Charité-University Medicine Berlin, Charitéplatz 1, 10117 Berlin, Germany; 4 Stresemannallee 3, 60596 Frankfurt, Germany; 5 Bahnhofsallee 3-4, 31134 Hildesheim, Germany

## Abstract

**Introduction:**

We used the data of the German biologics register RABBIT, a nationwide prospective cohort study, to investigate the risk of new or recurrent malignancy in patients with rheumatoid arthritis (RA) receiving biologics compared to conventional disease modifying anti-rheumatic drugs (DMARDs).

**Methods:**

The analysis was based on patients with RA enrolled in RABBIT at the start of a biologic or conventional DMARD therapy between 01 May 2001 and 31 December 2006. Incidences of first or recurrent malignancies were analysed separately. A nested case-control design was used to investigate the risk of developing a first malignancy. Matching criteria were: age, gender, follow-up time, disease activity score based on 28 joint counts (DAS28) at study entry, smoking status, and selected chronic co-morbid conditions (obstructive or other lung disease, kidney, liver or gastrointestinal disease, psoriasis).

**Results:**

A prior malignancy was reported in 122 out of 5,120 patients. Fifty-eight of these patients had received anti-TNFα agents, 9 anakinra, and 55 conventional DMARDs at study entry. In 14 patients (ever exposed to anti-TNFα: eight, to anakinra: one) 15 recurrent cancers were observed. The average time period since the onset of the first malignancy was nine years. Crude recurrence rates per 1,000 patient-years (pyrs) were 45.5 for patients exposed to anti-TNFα agents, 32.3 for anakinra patients and 31.4 for patients exposed to DMARDs only (Incidence rate ratio anti-TNFα vs. DMARD = 1.4, *P *= 0.6.). In patients without prior cancer, 74 patients (70% female, mean age: 61.3) developed a first malignancy during the observation. This corresponds to an incidence rate (IR) of 6.0/1,000 pyrs. Forty-four of these patients were ever exposed to anti-TNFα treatment (IR = 5.1/1,000 pyrs). In a nested case-control study comparing cancer patients to cancer-free controls, 44 of the cancer patients and 44 of the cancer-free controls were ever exposed to anti-TNFα agents (*P *= 1.0).

**Conclusions:**

No significant differences in the overall incidence of malignancies in patients exposed or unexposed to anti-TNFα or anakinra treatment were found. The same applied to the risk of recurrent malignancies. However, in particular this last finding needs further validation in larger data sets.

## Introduction

Patients with rheumatoid arthritis (RA) and other chronic inflammatory diseases are often subject to prolonged treatment with immunosuppressive drugs which modify the immunologic pathways involved in the pathogenesis of RA. Tumor necrosis factor alpha (TNFα) is among the cytokines that play a major role in the inflammatory process of rheumatic diseases. Its inhibition leads to substantial improvement in clinical signs and symptoms in a majority of patients. To date three different agents are available as monoclonal antibodies or receptor fusion antagonists of TNFα. The finding that TNFα is able to induce tumor cell apoptosis led it to be named TNF before its role in the inflammatory process was revealed [1]. TNFα or rather its nuclear factor-kappa B pathway acts as an early tumor suppressor [2]. This property led to concerns about a possibly increased risk of malignancies when drugs blocking TNFα will be used for long-term treatment.

These concerns were supported by two meta-analyses of randomized controlled trial data. In their first aggregate data meta-analysis of nine randomized controlled trials (RCTs) of anti-TNFα antibody therapies (infliximab and adalimumab) versus placebo in patients with rheumatoid arthritis, Bongartz et al. [3] found a significantly increased risk for malignancies in anti-TNFα versus placebo treated patients with a pooled odds ratio of 3.3 (95% CI: 1.2 to 9.1). In their second meta-analysis Bongartz et al. [4] found a higher malignancy risk also in patients treated with etanercept as compared to the control group, although the relative risk estimate did not achieve statistical significance (Hazard ratio (HR) of 1.84 [95% CI: 0.79 to 4.28]).

Considering the strict criteria for the inclusion of patients and the thorough monitoring process preceding controlled trials there might be an even higher risk when unselected RA patients are treated with anti-TNFα agents in daily rheumatologic care. Therefore, real-world data from studies systematically observing patients treated with these agents for long periods are of high importance.

Patients with prior malignancy are usually excluded from participation in RCTs and most clinical recommendations do not encourage treating these patients with anti-TNFα. However, this treatment might be the best therapeutic option for their inflammatory disease. Information regarding the safety of biologic agents prescribed to patients with prior malignancies is available only from two abstracts from the British Society of Rheumatology Biologics Register (BSRBR) [5,6], one of them indicating a possibly increased recurrence risk for melanoma [6].

According to the national recommendations of the German Society of Rheumatology biologic agents should be prescribed after failure of at least six months of treatment with two conventional DMARDs (including methotrexate (MTX)) alone or in combination [7].

The German biologics register RABBIT is an ongoing, nationwide prospective cohort study started in 2001 with the approval of the first biologic agents in Germany. It was established with the aim to assess the long-term safety of biologic agents including TNFα blockers. Time points of follow-up and assessments are identical for patients treated with biologic agents and for those under therapy with conventional DMARDs.

We used the data from RABBIT to investigate the frequency of developing a first malignancy in patients treated with anti-TNFα agents compared to those treated with conventional DMARDs and to study the risk of patients with a history of malignancy receiving anti-TNFα therapy.

## Materials and methods

### Patients

Patients aged 18 to 75 years meeting the American College of Rheumatology (ACR) criteria for RA are eligible to be enrolled in RABBIT at the start of treatment with a biologic agent or a conventional DMARD after failure of at least one other DMARD. Prior to enrollment all patients gave their informed consent. Patients enrolled between 01 May 2001 and 31 December 2006 (end of recruitment to this cohort) were included in the following analyses provided at least one follow-up visit and the baseline status regarding co-morbid conditions were available. Patients were followed up independent of any change in their treatment regimes. Information about patients who missed two or more consecutive follow-up visits was obtained by contacting the treating physicians, and if necessary the patients themselves, their relatives or the local health authorities to determine the patient's vital status. The reasons for dropout and the causes of death were ascertained. Details of inclusion criteria for RABBIT were previously reported [8,9]. The ethics committee of the Charité University School of Medicine, Berlin, approved the study protocol.

### Assessments

At baseline and at predefined time points of follow-up (3, 6, 12, 18, 24, 30, 36, 48, 60 months) rheumatologists assessed the clinical status including the components of the disease activity score based on 28 joint counts (DAS28) and reported treatment details and serious and non-serious adverse events according to the International Conference on Harmonization E2A guidelines [10]. All adverse events were coded using the Medical Dictionary for Regulatory Affairs (MedDRA) [11] by one of the authors (AS). Reported malignancies were considered as *events of interest*, and an additional query asking for diagnostic and treatment details and cancer history was sent to the reporting rheumatologist. Only in five cases we did not receive any further information. In 50% of the cases hospital discharge letters with the exact histopathologic results were sent to us.

At study entry rheumatologists reported co-morbidities for every patient on a list of 23 diseases which include among others: prior malignancy or lymphoma, chronic obstructive pulmonary disease (COPD), other chronic lung disease, chronic renal disease, chronic gastrointestinal disease, chronic liver disease, psoriasis, and chronic viral disease. Patients assessed their pain, general health, disability and socioeconomic status. The Hannover Functional Status Questionnaire (Funktionsfragebogen Hannover, FFbH) was used to assess disability. Scores are expressed as percentage of full function (range 0 to 100) and can be transformed into Health Assessment Questionnaire (HAQ) values [12]. Smoking habits were not assessed at baseline but only after 24, 48, 60 months. Since this resulted in a high percentage of missing smoking information we did not include smoking in the multivariate analyses. Nevertheless, in the nested case control study we were able to include smoking by using the missing information as one matching criterion.

### Statistical analysis

Prior malignancies and tumor recurrence. All patients meeting the study inclusion criteria were stratified by their prior malignancy status. For all patients prior malignancies were reported by the rheumatologist at study entry. Patients with or without prior malignancy were compared with respect to patient characteristics and treatment. In patients with a recurrent malignancy during the observation in RABBIT we analysed whether treatment was associated with recurrence. We defined recurrency as development of any cancer after a history of a prior malignancy, irrespective of the type of the recurrent tumor.

#### Tumor incidence

We analysed the tumor incidence during the observation period in all patients without prior malignancy. We included all types of cancer except for basal cell carcinomas. One M. Bowen was reported and included. There was no report of other carcinomas in situ. The observed number of incident cancers was compared with the expected number calculated from population data [13]. Cox regression was used to analyse the effects of treatment and to adjust for demographic and clinical data. Only exposure times after enrollment in the RABBIT register were taken into account. Patients were considered to be exposed to anti-TNFα treatment for the time period from the start of anti-TNFα treatment to the end of follow-up (*ever exposed*-approach). The same definition was used for anakinra exposure. Because of the applied *ever exposed*-approach patients could have been exposed to both, anti-TNFα agents and anakinra. Patients (or follow-up time of patients) not (yet) exposed to anti-TNFα or anakinra were considered as exposed to DMARDs only.

The following baseline characteristics were included in the risk assessment: age, gender, disease duration, rheumatoid factor, functional capacity (measured by the FFbH), selected previous treatment exposures (cyclosporine or azathioprine) [14], and co-morbid conditions.

Since nearly all (98.3%) of our patients were ever exposed to methotrexate and only a small minority (0.6%) to cyclosphosphamide we were not able to investigate a specific cancer risk associated with these agents. A preliminary analysis blinded for treatment assignment revealed associations between the frequency of cancer and COPD, chronic gastrointestinal diseases and chronic renal diseases. Other co-morbidities, such as chronic lung diseases in general, are known to be associated with increased cancer risk. Therefore, the following comorbid conditions were included in the Cox regression analysis: COPD, other chronic lung disease, chronic renal disease, chronic gastrointestinal disease, chronic liver disease, and psoriasis. Furthermore, we investigated the impact of exposure to anti-TNFα agents as well as the impact of long-term high disease activity (measured by time-averaged DAS28 scores) on the risk of cancer. For this analysis, the mean of all DAS28 scores measured more than six months before an event were included as time-dependent co-variables into the Cox regression analysis. Disease activity during the six months prior to a malignancy diagnosis was not considered since it may have been influenced by the carcinogenesis. For malignancies that developed within the first six months of observation, the DAS28 measured at study entry was used. On average 7% of the DAS28 values at follow-up were missing. To minimize possible bias missing values were imputed before the time-averaged scores were calculated. The expectation-maximisation (EM) algorithm most appropriate for approximately normally distributed variables such as the DAS28 was applied for estimation and imputation [15]. Calculations were performed using the SAS procedures MI and PHREG. A test based on the analysis of Schoenfeld residuals of Cox regression was used to investigate the invariance of the HR over time [16].

The control for confounding factors by Cox regression analysis may be insufficient since smoking could not be included and two possible risk factors found in our preliminary analysis (see above) were observed in less than 5% of the patients. Our statistical analysis plan therefore stipulated to perform a nested case control study as our main analysis of the risk of incident cancer. For each case with an incident cancer, a cancer-free control patient was selected who was compatible with the following matching criteria: gender, smoking status, and six co-morbid conditions (same as those used in the Cox regression). Cases with valid data of smoking status were matched to controls with the same smoking status and patients with missing information regarding smoking status were matched to controls who also had no smoking status data. After matching for eight categorical variables, a control patient was selected who fitted best to the case concerning age, follow-up time and DAS28 at baseline. Standardized Mahalanobis metric was used for measuring similarity. The availability of 4,923 possible controls permitted use of this detailed matching algorithm. Mc Nemar test was used to compare the numbers of patients exposed to biologics (anti-TNFα agents or anakinra) between patients and matched controls. For further comparisons within the nested case control study, paired t-test and Wilcoxon test were applied as appropriate. Chi-square test, t-test and Mann-Whitney test were used for statistical comparisons of patient's characteristics at baseline. *P*-values < 0.05 were considered statistically significant.

## Results

### Patient characteristics and treatment status at study entry

**Figure 1:**
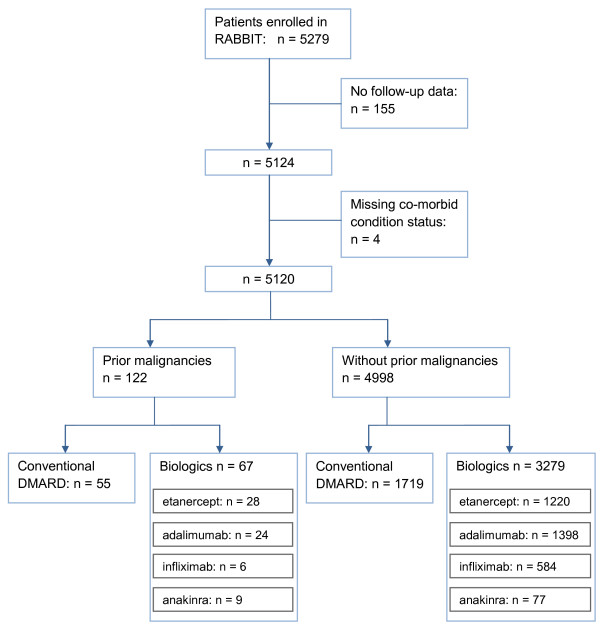
**Flow chart of patients included in the analysis**.

Between 01 May 2001 and 31 December 2006, 5,279 patients were enrolled in RABBIT. One hundred fifty-nine patients were excluded from this analysis because of missing follow-up information or missing co-morbid condition status (Figure 1). Their baseline characteristics (age, DAS28, function, co-morbidity status) were not statistically different from the remaining 5,120 patients. Those were stratified according to their prior malignancy status, and both groups were analysed separately. A total of 124 prior malignancies were found in 122 patients: 6 lymphomas (DMARDs: 2, anti-TNFα: 4), and 118 solid tumors (DMARDs: 54, anakinra: 9, anti-TNFα: 55)]. Patients with prior malignancies were significantly older (P < 0.001), had a lower functional capacity (56% of full function vs. 60% of full function) and a higher frequency of chronic gastrointestinal disease than patients without prior malignancy (Table 1). Within both strata, we found that patients receiving biologics had significantly more active disease, and were more limited in activities of daily living (FFbH). As reported previously, there were no significant differences in the clinical characteristics of patients receiving etanercept, adalimumab, or infliximab [17], whereas anakinra patients had more treatment failures with DMARDs and a lower functional capacity (FFbH) than anti-TNFα patients. Because of the differing modes of action and the differences in the clinical characteristics, separate results are provided in the following analyses for patients receiving anti-TNFα agents and patients receiving anakinra.

**Table 1:** Baseline characteristics of patients

	Patients with prior malignancy				Patients without prior malignancy				
	**Biologic**	**Control**	**Total**	***P *#**	**Biologic**	**Control**	**Total**	***P *#**	***P *##**

**n**	67	55	122		3279	1719	4998		
**Female n (%)**	45 (67.2)	41 (74.5)	86 (70.5)	0.43	2564 (78.2)	1353 (78.7)	3917 (78.4)	0.671	0.047
**Age (mean, SD)**	64.0 (9.0)	63.2 (7.7)	63.7 (8.4)	0.62	53.6 (12.3)	55.9 (11.5)	54.4 (12.1)	< 0.0001	< 0.001
**Disease duration (yrs), median (IQR)**	10 (6, 16.5)	7 (3, 13)	9 (4, 16)	0.02	9 (5, 17)	6 (2.5, 12)	8 (4, 15)	< 0.0001	0.286
**Time from prior cancer to study entry (yrs), median (IQR)**	5 (2, 9)	5 (3, 11)	5 (2, 10)	0.77					
**Follow-up time (yrs), median (IQR)**	2.1 (1.4, 3.0)	2.5 (1.0, 4.0)	2.1 (1.1, 3.1)	0.43	2.4 (1.4, 3.1)	2.5 (1.3, 3.3)	2.4 (1.3, 3.1)	0.081	0.611
**Rheumatoid factor positive n (%)**	53 (79.1)	46 (83.6)	99 (81.1)	0.64	2629 (80.2)	1225 (71.3)	3854 (77.1)	< 0.0001	0.327
**DAS28 (mean, SD)**	5.7 (1.3)	5.4 (1.1)	5.6 (1.2)	0.04	5.8 (1.3)	5.0 (1.3)	5.5 (1.3)	< 0.0001	0.282
**ESR (mm/h), median (IQR)**	38 (18, 51)	26 (15, 42)	32 (17, 50)	0.12	30 (16, 48)	22 (12, 38)	27 (14, 44)	< 0.0001	0.080
**CRP (mg/L), median (IQR)**	25 (10, 46)	15 (8, 30)	19 (9, 43)	0.07	17 (8, 38)	12 (5, 27)	15 (7, 34)	< 0.0001	0.146
**FFbH (mean, SD)**	52.1 (21.3)	59.9 (23.5)	55.7 (22.5)	0.02	57.0 (23.0)	66.8 (21.4)	60.4 (22.9)	< 0.0001	0.025
**Smoking ever n (%)**	23 (57.5)	15 (55.6)	38 (56.7)	1.00	878 (46.9)	473 (46.4)	1351 (46.7)	0.328	0.13
**No. of previous DMARDs (mean, SD)**	3.7 (1.5)	1.9 (1.0)	2.9 (1.6)	< 0.001	3.6 (1.4)	1.9 (1.1)	2.9 (1.5)	< 0.0001	0.656
**COPD n (%)**	3 (4.4)	6 (10.9)	9 (7.3)	0.30	163 (5)	81 (4.7)	244 (4.9)	0.685	0.205
**Chronic renal disease n (%)**	4 (5.9)	1 (1.8)	5 (4.1)	0.38	134 (4.1)	29 (1.7)	163 (3.3)	< 0.0001	0.603
**Chronic lung disease n (%)**	5 (7.4)	1 (1.8)	6 (4.9)	0.22	93 (2.8)	29 (1.7)	122 (2.4)	0.012	0.129

**Chronic gastrointest. disease n (%)**	**12 (17.6)**	**5 (9.1)**	**17 (13.9)**	**0.2**	**281 (8.6)**	**138 (8.0)**	**419 (8.4)**	**0.510**	**0.047**

Values are means and standard deviations if not otherwise specified. IQR = inter quartile range; FFbH = Hannover Functional Status Questionnaire measuring functional capacity in percent of full function; DAS28: disease activity score based on 28 joint counts;

# = *P*-value for comparison between biologic and control group within strata according to prior malignancy status, ## = comparison between strata

Patients with a prior malignancy were insignificantly less frequently treated with anti-TNFα agents or anakinra at inclusion than patients without prior malignancy (Figure 1, Table 1). The adjusted OR to receive biologics (adjusted for age, sex, disability, disease activity) for patients with prior malignancies compared to those without was 0.7 (95%CI: 0.5 to 1.1). Detailed information including the exact type of malignancy was reported in 54 of the 124 prior malignancies. We found some differences regarding the spectrum of those malignancies in anti-TNFα vs. DMARD treated patients: At study entry all nine cases with prior prostatic cancer were treated with biologics (seven with anti-TNFα and two with anakinra) whereas three patients with prior bladder cancer were found in the DMARD treated group and one patient was treated with anakinra. Patients with prior breast cancer were less frequently treated with biologics (n = 11) than with DMARDs (n = 14) at inclusion. The time between onset of the prior malignancy and study entry did not differ between the treatment groups. The median time was five years (IQR: 2 to 9) for patients receiving biologics (anti-TNFα: four years (2 to 10); anakinra: six years (5 to 9)) and five years (3 to 11) for patients receiving conventional DMARDs (*P *= 0.77). In 28 (45.9%) of the patients treated with biologics (27 with anti-TNFα and 1 with anakinra) and in 22 (40.7%) patients in the DMARD group the time since the last tumor diagnosis was less than five years when treatment with the respective agent started.

### Recurrence of a prior malignancy

During follow-up 15 recurrent cancers were observed in 14 patients including 14 recurrences of the same type and site as the prior tumor and one metastasis of unknown origin (Table 2). Nine recurrences were seen in eight patients under treatment with anti-TNFα agents, one in an anakinra patient and five in patients exposed to DMARDs only. The corresponding crude incidence rates were 45.5 (95%CI: 20.8 to 86.3)/1,000 patient years (pyrs) for patients receiving anti-TNFα agents, 32.3 (95%CI: 0.8 to 179.7)/1,000 pyrs for patients treated with anakinra and 31.4 (95%CI: 10.2 to 73.4)/1,000 pyrs for DMARD treated patients (incidence rate ratio anti-TNFα agents vs. DMARDs: 1.4 (95% CI: 0.5 to 5.5) *P *= 0.63).

**Table 2:** Recurrence of prior malignancy by type and treatment

		Ever exposed to		
	**Total**	**Anti-TNFα**	**Anakinra**	**Conventional DMARD only**

**N with prior malignancy**	122	72	11	43
**Patient-years of follow-up**	379	198	31	159
**Recurrent malignancies**	15	9 (5 f, 4 m)	1 (m)	5 (4 f, 1 m)
**Breast cancer**	5	4 (f)	-	1(f)
**Lung cancer**	3	1 (m)	1 (m)	1 (f)
**Bladder cancer**	2	1 (m)^#^	-	1 (f)
**Liposarcoma**	1	1 (m)	-	-
**Melanoma**	1	1 (f)		
**Signet-ring cell carcinoma**	1	-	-	1 (f)
**Testicular cancer**	1	1 (m)^#^	-	-
**Metastasis of unknown origin**	1			1 (m)

M = male, f = female, ^#^testicular cancer and bladder cancer in one patient

The mean time span between the prior tumor and the diagnosis of the new tumor was 9.5 (SD: 7.8), 9.1, and 9.2 (8.8.) years for patients exposed to anti-TNFα agents, anakinra, or conventional DMARDs, respectively. Three patients developed a recurrent cancer less than five years after the previous cancer (two in the anti-TNFα treated group, one in the DMARD group).

Four of the five patients who were treated with conventional DMARDs only and experienced a recurrence of their prior malignancy died (signet-ring-cell carcinoma, metastasis of unknown origin, breast cancer, lung cancer). One out of the eight patients under treatment with anti-TNFα died (breast cancer), and the one patient under treatment with anakinra (lung cancer) died. All other patients with recurrences were still alive at the time of the analysis.

### Incidence of tumors in patients without a prior malignancy

#### Comparison of the tumor incidence with the general population

**Figure 2:**
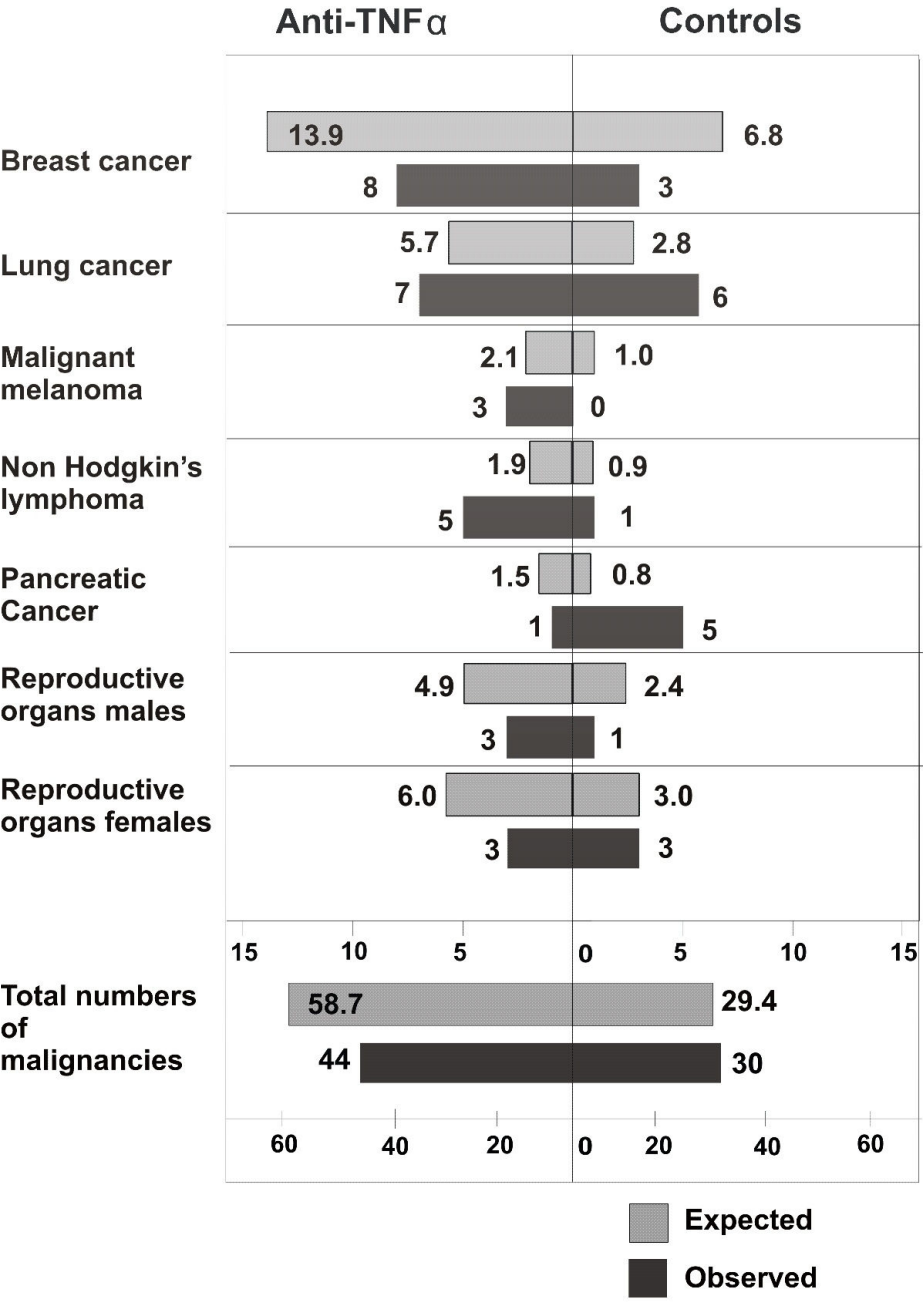
**Observed numbers of cancers and expected numbers from the general population, standardized by age and sex**.

Seventy-four patients among the 4,998 patients who did not have a prior malignancy developed an incident tumor. This is an overall incidence rate of 6.0 per 1,000 pyrs [95% CI: 4.7 to 7.6]. The figures were 5.1 per 1,000 pyrs (95% CI: 3.7 to 6.9) for patients exposed to anti-TNFα, 7.2 per 1,000 pyrs (95% CI: 2.4 to 16.9) for patients exposed to anakinra and 8.4 per 1,000 pyrs (95% CI: 5.7 to 12.0) for patients exposed to conventional DMARDs. For some of the cancer sites the observed incidence rates in both groups were lower than the age and sex adjusted rates as expected from the general population (for example, breast, male and female reproductive organs and colon cancer) [13] (Figure 2). Higher rates were observed for non-Hodgkin's lymphoma in patients exposed to biologics and for pancreatic cancer in the group not exposed to biologics. None of the site specific differences were statistically significant when the *P*-values were adjusted for repeated significance testing. Taking into account all malignancies, the number of observed cancers in patients exposed to anti-TNFα agents was non-significantly lower than the expected number from the general population (standardized incidence rate ratio: 0.75, 95% CI: 0.54 to 1.01). No difference was found for patients not exposed to biologics.

#### Comparison of patients with and without incident tumors in patients with no prior cancer

Overall, patients who developed malignancies during the study period had more co-morbidities than those who did not (mean = 2.5 (SD = 2.1) vs. 1.7(1.9)). Higher rates were observed for COPD (11/74 (14.9%) vs. 223/4924 (4.5%), *P *< 0.0001), chronic gastrointestinal diseases ((13/74 (17.6%) vs. 406/4924 (8.2%), *P *= 0.008), and chronic renal diseases (4/74 (5.4%) vs. 159/4924 (3.2%), *P *= 0.22). Furthermore, site specific associations were observed for gastric/colorectal cancer in 2/419 patients with a chronic gastrointestinal disease vs. 3/4579 in the remaining patients (*P *= 0.06), and for bladder cancer/renal cancer in 1/163 patients with chronic renal diseases vs. 3/4835 in the remaining patients (*P *= 0.12).

Crude cancer incidence rates were therefore higher in patients with specific comorbid conditions but also in those with a highly active disease (Table 3).

**Table 3:** Crude incidence rates of malignancies

	n of CA	n	pyrs	IR
**Characteristics at baseline**				
Age (per 10 yrs increase)				
Female	52	3,917	9,687	5.4
Male	22	1,081	2,554	8.6
Co-morbid conditions:				
COPD				
No	63	4,754	11,677	5.4
Yes	11	244	564	19.5
Gastrointestinal disease				
No	61	4,579	11,153	5.5
Yes	13	419	1,088	11.9
Renal disease				
No	70	4,835	11,884	5.9
Yes	4	163	357	11.2
				
**Characteristics at follow-up**				
DAS28				
<3.2	6	787	2,165	2.8
3.2 to 5.1	34	2,823	7,341	4.6
>5.1	34	1,388	2,735	12.4
				
**Ever exposed to**				
Conventional DMARDs only	30	1,684 ^#^	3,561	8.4
Anti-TNF agents	44	3,651 *	8,558	5.1
Anakinra	5	247 ^$^	690	7.2

CA: malignancies, n = number of cases, pyrs = follow-up time in patient years, IR = crude incidence rate per 1,000 pyrs.

^#^35 of the patients in the DMARD cohort were exposed to anti-TNF agents before inclusion in the study. According to the ever exposed approach their follow-up time was assigned to the anti-TNF treated group.

* Patients included in this group are patients who were included in the study with the start of an anti-TNF agent (n = 3,201) and patients who changed during follow-up from DMARD or anakinra treatment to anti-TNF treatment

^$^223 of the 247 pts were exposed to anakinra and anti-TNF agents, they experienced five malignancies.

The univariate analysis showed that patients with a very active disease (DAS28 >5.1, mean: 5.93) during follow-up had a two times higher cancer risk than those with low disease activity (DAS28 <3.2, mean: 2.75) (Table 4).

**Table 4:** Hazard ratios of developing a malignancy

	Univariate Cox regression			Multivariate analysis		
	HR	95% CI	*P*	adjusted HR	95% CI	*P*
**Characteristics at baseline**						
Age (per 10 yrs increase)	1.82	1.44 to 2.31	< 0.0001	1.71	1.35 to 2.17	< 0.0001
Male vs. female	1.61	0.98 to 2.65	0.062	1.47	0.89 to 2.43	0.13
Co-morbid conditions:						
COPD	3.64	1.92 to 6.91	< 0.0001	2.63	1.37 to 5.04	0.004
Gastrointestinal disease	2.19	1.20 to 3.98	0.010	1.81	0.99 to 3.30	0.0534
Renal disease	1.93	0.70 to 5.28	0.20			
						
**Characteristics at follow-up**						
DAS28 (per unit increase)	1.24	1.02 to 1.50	0.034			
DAS28						
< 3.2	*Referent*					
3.2 to 5.1	1.28	0.54 to 3.06	0.58			
> 5.1	2.00	0.82 to 4.86	0.13			
						
**Ever exposed to**						
Conventional DMARDs only	*Referent*			*Referent*		
Anti-TNF agents	0.61	0.39 to 0.97	0.039	0.70	0.44 to 1.12	0.133
Anakinra	1.16	0.47 to 2.89	0.75	1.39	0.56 to 3.48	0.480

In the multivariate analysis the development of an incident tumor was strongly associated with age (HR = 1.71; 95% CI: 1.3 to 2.2 per 10 years increase in age, *P *< 0.0001) and COPD (HR = 2.63; 95% CI: 1.4 to 5.0, *P *= 0.004) (Table 4). A higher cancer risk was also observed for patients with chronic gastrointestinal diseases whereas no significant associations were found for other co-morbid conditions (other chronic lung diseases, psoriasis, chronic liver disease) or for gender (HR (males vs. females) = 1.46; 95% CI: 0.9 to 2.4, *P *= 0.14). Likewise, associations for patients ever exposed to cyclosporine (n = 582, *P *= 0.24) or azathioprine (n = 599, *P *= 0.32) were not statistically significant.

In patients exposed to anti-TNFα agents we observed a non-significantly decreased risk for developing a malignancy compared to patients treated with conventional DMARDs (adjusted HR = 0.70; 95% CI: 0.44 to 1.12, *P *= 0.13).

### Time dependency of the hazard risk in patients without prior cancer

The mean time until the onset of the malignancy was 25.0 (16.8) months for patients exposed to anti-TNFα agents, 14.8 (9.1) for anakinra and 17.4 (15.7) months for patients not exposed to biologics. Ten of the 44 malignancies of anti-TNFα-exposed patients developed in the first year compared to 15 of 30 malignancies in non-exposed patients. This corresponds to adjusted hazard ratios of 0.4 for the first and 1.0 for the second to fourth year. We analysed this possible time trend in the hazard ratio by means of an analysis of standardized Schoenfeld residuals of the Cox regression. The trend was, however, not statistically significant (*P *= 0.13).

### Nested case-control study

The main analysis to assess the risk of developing an incident tumor under treatment with anti-TNFα agents was conducted as a nested case control study. Each case with an incident malignancy was matched to one control patient without malignancy (see Methods). Due to the high number of possible matching partners, matching was successful for all parameters involved. Cases with incident malignancy had lower baseline functional capacity than those without cancer (Table 5). There was no significant difference concerning treatment exposure: Forty-four (59.5%) of the cases and 45 (60.8%) of the controls had ever been exposed to biologics. The numbers of cases ever exposed to etanercept, adalimumab, infliximab, or anakinra (n = 22; n = 20; n = 16; n = 5, respectively) did not differ significantly from the numbers of controls ever exposed to these therapies (n = 27; n = 24; n = 10; n = 5, respectively). A separate analysis of malignancies observed in the first year (anti-TNFα exposed 10/25 cases vs. 11/25 controls) and in the second to fourth year (anti-TNFα exposed: 34/49 cases vs. 33/49 controls) did not show any significant difference between the groups or a significant time trend.

**Table 5:** Patient characteristics of cases and matched controls

	Cases (with incident malignancy)	Matched controls	P
N	74	74	
**Characteristics at study entry**			
Females^# ^n (%)	52 (70.3)	52 (70.3)	n.a.
Age^# ^(mean, SD)	61.3 (8.9)	61.4 (8.5)	0.97
Observation time^# ^(years) (median, IQR)	2.9 (1.8, 4.0)	2.9 (1.6, 3.9)	0.25
DAS28^# ^(mean, SD)	5.6 (1.0)	5.7 (1.0)	0.31
Smoking status^#^			
Nonsmoker	16 (21.6)	16 (21.6)	n.a.
Smoker n (%)	19 (25.7)	19 (25.7)	
Unknown status n (%)	39 (52.7)	39 (52.7)	
Disease duration (years) (median, IQR)	7 (3,14)	9 (5,16)	0.22
Functional status, FFbH (mean, SD)	57.1 (22.3)	63.2 (22.6)	0.058
**Characteristics at follow-up**			
DAS28 (mean, SD)^$^	5.0 (1.2)	4.9 (1.2)	0.41
Ever exposed to biologics n (%)	44 (59.5)	45 (60.8)	1.0
Ever exposed to anti-TNFα agents ^&^	44 (59.5)	44 (59.5)	1.0
Ever exposed to anakinra^&^	5 (6.8)	5 (6.8)	1.0
**Among them**			
Cases with solid tumors (n = 68)			
Ever exposed to biologics	39 (57.4)	43 (63.2)	0.56
Ever exposed to anti-TNFα agents	39 (57.4)	42 (61.8)	0.70
Ever exposed to anakinra	5 (7.4)	5 (7.4)	**1.0**
			
**Cases with non-Hodgkin's Lymphoma (n = 6)**			
**Characteristics at study entry**			
**Females^# ^n(%)**	5 (83.3)	5 (83.3)	**n.a.**
**Age^# ^(mean, SD)**	65.2 (6.5)	66.8 (8.0)	**0.13**
**Disease duration**	5.5 (4,13)	7 (5,11)	**0.53**
**Functional status, FFbH (mean, SD)**	52.8 (31.4)	54.2 (27.5)	**0.90**
**DAS28^# ^(mean, SD)**	5.9 (0.6)	6.2 (0.5)	**0.29**
**Characteristics at follow-up**			
**DAS28 (mean, SD)^$^**	5.2 (0.7)	5.5 (0.7)	**0.14**

**Ever exposed to anti-TNFα agents**	**5 (83.3)**	**2 (33.3)**	**0.38**

^# ^matching criteria (further matching criteria not shown in the table = COPD, other chronic lung disease, chronic renal disease, chronic gastrointestinal disease, chronic liver disease, and psoriasis)

^&^five cases and four controls received anti-TNFα agents and anakinra (at different points in time)

^$^cases = mean of DAS28 values over time points until six months prior to the cancer diagnosis, matched controls: mean of DAS28 values over time points of the corresponding case

An insignificantly higher rate of exposure to anti-TNFα agents was found in patients who developed non-Hodgkin's lymphoma.

## Discussion

First, in patients with prior malignancy we did not find a significant increase in the risk of recurrent tumors under treatment with anti-TNFα agents compared to conventional DMARDs, even though there was a higher recurrence rate under anti-TNFα treatment (IRR = 1.4, *P *= 0.6).

Second, patients without prior malignancy did not have higher rates of incident tumors when they were exposed to biologics compared to unexposed patients.

The strength of our study is that all data on incident and recurrent tumours originate from a prospective, closely monitored observational cohort study established for the purpose of pharmacovigilance. Data were collected in an identical manner and by the same physicians for patients treated with biologic agents or with conventional DMARDs. Due to stringent and close monitoring, drop-out rates were less than 5% per year. Additionally, for all patients lost to follow-up the vital status was ascertained or, if appropriate, the cause of death.

To investigate the occurrence of incident malignancies, we followed three different methodological approaches: a nested case-control study, a multivariate Cox regression and a comparison with population data.

Of note, we excluded basal cell carcinoma from all types of our analysis since no age and sex specific population rates were available.

The design of the nested case control study allowed us to adjust for differences in clinical and demographic parameters (for example, selected co-morbidities, smoking status) which are related to treatment assignment but which are also associated with the risk of cancer. We therefore consider the nested-case control analysis our central assessment of the risk of incident malignancies under treatment with anti-TNFα agents. However, since only a very small proportion of the patients were included in the nested case control analysis and therefore information of a large number of patients was not used, we decided to apply a multivariate Cox regression analysis in addition.

A potential weakness of this study is that we only investigated the overall cancer risk. The risk for site-specific cancers has not been analyzed due to the limited numbers of events. In addition, due to the relatively short time of observation no conclusions can be drawn beyond the scope of four years of exposure.

Limitations of the analysis of tumor recurrence include the relatively small sample size and the fact that physicians were less likely to prescribe cytokine inhibitors for patients with prior malignancy than for those without such a history. Furthermore, our data indicate that physicians might have made different treatment decisions for patients with different prior tumors. We therefore must exhibit caution in drawing firm conclusions.

The same applies for the interpretation of the data for anakinra treated patients: only a few patients in the cohort were ever treated with anakinra, most of them were also exposed to anti-TNFα agents. Therefore, malignancies occurring in this group must be interpreted carefully, taking patient selection (also seen in differing baseline characteristics) into account.

Our results differ from those reported by the British Society for Rheumatology Biologics Registry (BSRBR) [6]. Dixon and co-workers analyzed 177 patients with prior malignancies treated with anti-TNFα agents with a median follow-up of three years and found no increased risk for recurrent malignant diseases compared to 118 patients with prior malignancies treated with conventional DMARDs and followed-up for 1.9 years. Their crude incidence rates were 25.3 and 41.9 per 1,000 patient-years for patients treated with anti-TNFα agents and conventional DMARDs, respectively, compared to 45.5 and 31.4 per 1,000 patients-years in our study. In an earlier analysis of the BSRBR [5] a total of 154 patients in the anti-TNFα cohort had a previous malignancy and six (4%) developed a new malignancy. However, of these malignancies only one was a local recurrence which is in contrast to our findings. In our study, in 14 out of the 15 recurrences the observed malignancies were *true *recurrences of the prior tumor with the same type and site. Only in one of our patients with pulmonary and bone metastases the origin of the malignancy remained unknown. This patient had a history of testicular cancer 20 years before.

Our investigations regarding the risk of developing an incident malignancy were motivated by two meta-analyses of randomized controlled trials which suggested an increased risk of malignancies associated with the treatment with one of the TNFα blocking agents: adalimumab, infliximab, or etanercept [3,4]. The results of these meta-analyses are in contrast with those from observational cohort studies or national cancer registries, in which such an increased risk was not observed [18,19]. The methodological weaknesses of the first meta-analysis investigating the risk for treatment with adalimumab or infliximab have already been discussed elsewhere [20]. However, the repeated finding of an increased cancer risk also in patients treated with etanercept [3,4] requires further research, even if it did not achieve statistical significance. These meta-analyses support the *early mobilization hypothesis *implying a high risk within the first months of treatment with anti-TNFα agents.

However, our results for the first year are in contrast to the meta-analyses reported by Bongartz et al. [3,4]. In the first year of treatment patients receiving biologics had a lower risk for developing an incident malignancy than those receiving conventional DMARDs. This may be due to a selection bias evoked by the screening process for malignancies following the physician's decision that a patient should receive biologic treatment. Screening for latent TB by chest x-ray as recommended in guidelines [21] may reveal asymptomatic lung cancers and only those patients who screen negative for current malignancies will receive biologic treatment and be included in the biologics group in the RABBIT register. No such general screening occurs in patients who will receive a new DMARD therapy. Therefore the rate for malignancies in the DMARD group represents the *true *unselected rate that can be expected for RA patients under conventional DMARD treatment. Indeed this concept is supported by our findings that the observed number of cancer cases in the DMARD treated group (n = 30) was equal to the expected number (n = 30.8), whereas the observed number in the biologics group (n = 44) was lower than what would have been expected based on the rates from the general population (n = 64.3).

In contrast to an increased risk, it is also possible that inhibition of TNFα has beneficial or even preventive effects regarding cancer. TNFα is important in all steps of cancer development, for example, initiation, promotion, and survival. Elevated levels of TNFα are linked to a poor prognosis and increased invasiveness in certain human cancers [22,23]. However, the results of first trials to treat breast or ovarian cancer with TNFα inhibitors have been, so far, inconclusive [24,25]. The increased risk of non-Hodgkin's lymphoma in RA patients treated with biologic agents is well established [26,27] and has been shown to be strongly associated with long-term high disease activity [28] which is more likely in the history of patients subsequently treated with biologic agents. Nevertheless, in our nested case control study where we controlled for disease activity and duration we still found a higher proportion of anti-TNFα exposure in patients with incident non-Hodgkin's lymphomas vs matched controls. This difference was not found for solid tumors.

## Conclusions

Our data add to the growing evidence of no overall increased cancer risk for patients treated with anti-TNFα agents during the first years of treatment. This does not preclude that there may be an increased risk for specific cancer types such as lymphoma or skin cancer.

Taking the limitations of the currently available evidence into account there is a need for further large-scale prospective studies investigating risk modifications for different cancer sites as well as investigating the cancer risk after long-term exposure to biologic agents above four years.

Further, this study provides first but limited evidence regarding the risk of patients with a prior malignancy treated with anti-TNFα agents. The finding of an insignificantly increased risk of recurrence under anti-TNFα treatment supports the current practice of carefully balancing treatment decisions in these patients.

## Abbreviations

ACR: American College of Rheumatology; BSRBR: British Society of Rheumatology Biologics Register; CI: confidence interval; COPD: chronic obstructive pulmonary disease; DAS28: disease activity score based on 28 joint counts; DMARDs: disease modifying anti-rheumatic drugs; FFbH: Hannover Functional Status Questionnaire (Funktionsfragebogen Hannover); HAQ: Health Assessment Questionnaire; HR: hazard ratio; IQR: interquartile range; IR: incidence rate; IRR: incidence rate ratio; MedDRA: Medical Dictionary for Regulatory Affairs; OR: odds ratio; pyrs: patient-years; RA: rheumatoid arthritis; RABBIT: (German biologics register) acronym for: rheumatoid arthritis observation of biologic therapies; RCT: randomized controlled trial; SD: standard deviation; TB: tuberculosis; TNFα: tumor necrosis factor alpha.

## Competing interests

The authors declare that they have no competing interests.

## Authors' contributions

AS, JL and AZ had full access to all of the data in the study and took responsibility for the integrity of the data and the accuracy of the data analysis. AZ, JL and AS determined the study concept and design. AS, GRB, BK-L and WD acquired the data. AS, JL and AZ analysed and interpreted the data. AS, JL and AZ drafted the manuscript. RR, GRB, BK-L, WD and FH critically revised the manuscript for important intellectual content and final approval. FH and JL did the statistical analysis. AZ obtained the funding and supervision. AS supervised adverse events reporting, verifications and MedDRA coding. All authors gave final approval of the version to be published.
